# Application of Real-time Elastography Ultrasound in the Diagnosis of Axillary Lymph Node Metastasis in Breast Cancer Patients

**DOI:** 10.1038/s41598-018-28474-y

**Published:** 2018-07-06

**Authors:** Yanjun Xu, Xiaojun Bai, Yini Chen, Lixin Jiang, Bing Hu, Bin Hu, Li Yu

**Affiliations:** Department of Ultrasound in Medicine, Shanghai Jiao Tong University Affiliated Sixth People’s Hospital, Shanghai Institute of Ultrasound in Medicine, Shanghai, 200233 China

## Abstract

The pathological status of axillary lymph nodes (ALN) plays a critical role in the staging and treatment of patients with breast cancer. Therefore, differential diagnosis of metastatic ALN is highly desirable in the clinic. We used real-time elastography (RTE) and gray-scale ultrasound to generate a new scoring system for determining ALN status and estimate their performance of this system. Ninety-seven ALNs were examined by both gray-scale ultrasound and RTE. The performance of gray-scale ultrasound was evaluated by the sum of scores according to its features. RTE images were determined by a modulated scoring system based on the percentage and distribution of hypoechoic cortex regions in the ALNs. Strain ratio was also calculated. Diagnostic performance was obtained by receiver operating characteristic curve analysis with pathologic findings used as the reference standard. The sensitivity, specificity and accuracy were 92%, 73% and 83%, respectively, for gray-scale ultrasound; 78%, 93%, 86%, respectively, for RTE; 88%, 96% and 92%, respectively, for the combined evaluation (AUC = 0.963), and 87%, 76% and 81%, respectively, for strain ratio. Gray-scale ultrasonography had a better sensitivity than RTE (92% vs 78%, *P* = 0.039), while the specificity for RTE was superior to that of gray-scale ultrasonography (93% vs 73%, *P* = 0.012). In conclusion, RTE showed a high specificity for evaluating the ALN status and may improve the diagnostic accuracy when combined with gray-scale ultrasound.

## Introduction

The status of axillary lymph nodes is one of the most important prognostic factors for evaluating breast cancer, and it is also mandatory for proper treatment planning^1^. Traditionally, the axillary lymph node status is used to be determined by the histological results of axillary lymph node dissection (ALND). Due to the inevitable complications induced by the ALND, such as axillary swelling, nerve injury, and infection, the sentinel lymph node biopsy (SLNB) has gradually replaced ALND in recent years^2–7^. However, SLNB is still an invasive method, and the false negative results cannot be avoided. On the other hand, the edema induced by the SLNB of the axillary area and arm induced by SLNB is still a post-operative complication that the clinicians are concerned about. Ultrasound is a non-invasive method and it plays an important role in the preoperative evaluation of the axillary lymph nodes in breast cancer patients^8–10^. However, the accuracy of conventional gray-scale ultrasound (US), which focuses on the morphological features of the lymph nodes, is still moderate. A study on breast cancer patients with palpable and non-palpable axillary lymph nodes showed that the sensitivity and specificity of ultrasonography varied from 66.1 to 87.1% and from 44.1 to 97.9%, respectively^11^. While other research showed an 88.6% sensitivity, a 35.9% specificity, a 47.0% positive predictive value and an 83.0% negative predictive value^12^. With the development of colour Doppler flow imaging and high-frequency US probes, wide ranges of sensitivity and specificity of 52.6–86.49% and 71–100%, respectively, were obtained^13–15^.

Sonoelastography or ultrasound elastography is an imaging technique that utilizes the long-established clinical concept that malignant lesions are often stiffer than normal tissues. The stiffness, which is another important biomechanical characteristic of the tissue that is not be reflected on the gray-scale sonograms, can be estimated by the sonoelastography. The elastograms established by the real-time elastography (RTE) technique are colour-coded maps indicating the elasticity of different portions of the target tissue according to the analysis of changes in radiofrequency impulses changes before and after the manual rhythmical compression along the radiation axis. In RTE, elastograms are generated simultaneously with the gray-scale sonograms in order to ensure that images of the same node are precisely obtained^16–18^.

Though sonoelastography has been applied for the diagnosis of pathological changes in many kinds of superficial tissues, such as breast, thyroid, muscle, parotid, cervical lymph nodes and skin, there is relatively little documented literature about the use of sonoelastography for evaluating axillary lymph nodes in breast cancer patients^19–26^.

In the present study, both routine gray-scale ultrasound and modified RTE were used to evaluate the condition of the ALNs. Gray-scale sonograms were evaluated based on a sum of scores according to five criteria including the short-axis diameter, shape, hilum (present or absent), cortical thickness and border (regular or irregular) of the ALNs. RTE images were evaluated with a modulated scoring system based on the percentage and distribution of the hypoechoic cortex regions with a visible hilum in ALN or all the hypoechoic lymph nodes with an absent hilum. The strain ratio for the axillary lymph nodes was also calculated.

## Results

### Pathological Diagnoses

The pathological results of 97 axillary lymph nodes were reported, and 52 (53.6%) were determined to be metastatic, while the other 45 (46.4%) nodes were determined to be benign. The workflow was shown in Fig. S1. The US characteristics of the lymph nodes are shown in Table 1.

**Table 1:** Results of Examined Lymph Nodes According to the Criteria.

Criteria	Benign Lymph Nodes (n = 45)	Metastatic Lymph Nodes (n = 52)	*P* value
**Gray-scale ultrasound**			
Short-axis diameter			= 0.014
<7 mm	21 (47)	14 (27)	
≥7 mm	24 (53)	38 (73)	
L/S^a^			= 0.066
>2	23 (51)	17 (33)	
≤2	22 (49)	35 (67)	
H/L^b^			<0.01
>0.5	32 (78)	16 (31)	
≤0.5	13 (22)	36 (69)	
Cortex thickness			<0.01
<3 mm	37 (82)	23 (44)	
≥3 mm	8 (18)	29 (56)	
Border			= 0.014
Regular	25 (56)	16 (31)	
Irregular	20 (44)	36 (69)	
**Elastography score**			
1	16 (35)	0	
2	26 (58)	11 (21)	
3	3 (7)	14 (27)	
4	0	20 (38)	
5	0	7 (14)	
Strain ratio	2.35 ± 1.80 (0.51–6.31)	12.64 ± 5.30 (7.6–39.9)	<0.01
<2.62	33 (73)	9 (17)	
≥2.62	12 (27)	43 (83)	

Note-Numbers in parentheses are percentages. ^a^L/S indicates long-axis diameter to short-axis diameter ratio. ^b^H/L indicates hilum diameter to long-axis diameter of lymph node.

### Gray-scale Ultrasound Characteristics

The gray-scale characteristics of all the lymph nodes are shown in Table 1. The short-axis diameter, long-to-short axis (L/S) ratio, cortical thickness, border, H/L (H/L indicates the ratio of the hilum diameter to the long-axis diameter of the lymph node) and hilum features were measured in the present study to evaluate the lymph node status. The short-axis diameter, cortical thickness, border, and hilum features showed the substantial differences between benign and malignant lymph nodes. The sensitivity, specificity, and accuracy of H/L were 69%, 78%, and 70%, respectively. Cortical thickness had a relatively high specificity (82%), while the border and H/L had relatively low sensitivities (69% and 69%). When using 2 as the threshold value for the L/S ratio, there was no significance difference between the benign and metastatic lymph nodes (*P* = 0.066). According to the ROC curve analysis, the cutoff value was between 2 and 3 for gray-scale ultrasound. The nodes that were scored 1 or 2 were considered benign, and those that were scored 3, 4 or 5 were considered metastatic based on gray-scale ultrasound. When using the abovementioned cutoff value, the sensitivity, specificity, PPV, NPV and accuracy for gray-scale ultrasound were 92%, 73%, 80%, 89% and 83% respectively (Table 2).

**Table 2:** Evaluation of diagnostic performance of different methods with cutoff points for diagnosis of axillary lymph nodes.

Cutoff Points	Sensitivity (%)	Specificity (%)	Positive Predictive Value (%)	Negative PredictiveValue (%)	Accuracy (%)
3 for gray-scale ultrasound	92 (81, 96)	73 (59, 84)	80 (69, 88)	89 (75, 96)	83 (75, 90)
3 for real-time elastography	78 (66, 88)	93 (82, 98)	93 (82, 96)	79 (67, 88)	86 (77, 91)
6 for combined evaluation	88 (77, 95)	96 (85, 99)	96 (86, 99)	88 (76, 94)	92 (85, 96)
2.62 for strain ratio	87 (74, 94)	76 (61, 87)	80 (68, 89)	83 (68, 93)	81 (72, 88)

Note-Numbers in parentheses are 95% confidence intervals (CIs).

### Real-time Elastography Characteristics

**Figure 1:**
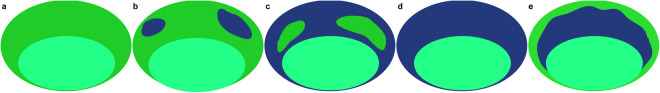
Schematic RTE scoring of hypoechoic regions in the ALNs with hila (Pattern I). Elastographic patterns were determined based on the distribution and percentage of lymph node area with a high elasticity (hard): (**a**) Green portion occupying almost all of the cortex; (**b**) Blue portion occupying less than 50% of the cortex; (**c**) Blue portion occupying more than 50% of the cortex, with a scattered green portion; (**d**) Blue portion occupying almost all of the cortex; (**e**) Blue portion occupying almost all of the cortex, with a green ring on the edge of the node.

**Figure 2:**
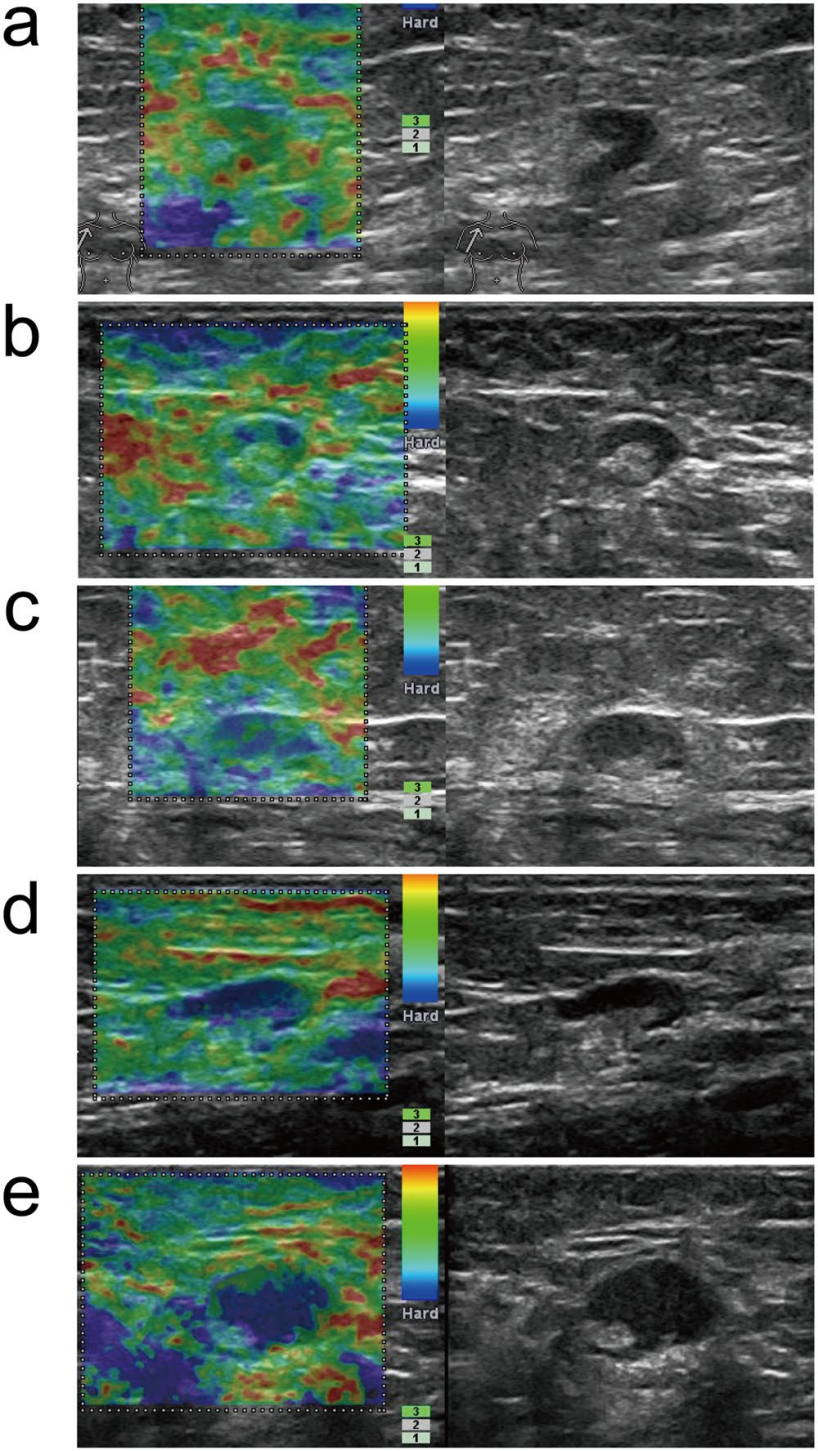
Gray-scale-RTE dual-mode ultrasonogram of Pattern I ALNs. Images present the general appearance of lesions for RTE scores of (**a**) 1, (**b**) 2, (**c**) 3, (**d**) 4, and (**e**) 5 of the lymph nodes with hila: (**a**) Green portion occupying almost all of the cortex; (**b**) Blue portion occupying less than 50% of the cortex; (**c**) Blue portion occupying more than 50% of the cortex, with a scattered green/red portion; (**d**) Blue portion occupying almost all of the cortex; (**e**) Blue portion occupying almost all of the cortex, with a green/red ring on the edge of the node.

**Figure 3:**
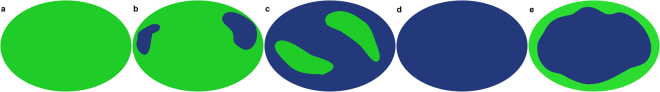
Schematic RTE scoring of all ALNs without hila (Pattern II). Elastographic patterns were determined based on the distribution and percentage of lymph node area with a high elasticity (hard): (**a**) Green portion occupying almost all of the cortex; (**b**) Blue portion occupying less than 50% of the cortex; (**c**) Blue portion occupying more than 50% of the cortex, with a scattered green portion; (**d**) Blue portion occupying almost all of the cortex; (**e**) Blue portion occupying almost all of the cortex, with a green ring on the edge of the node.

**Figure 4:**
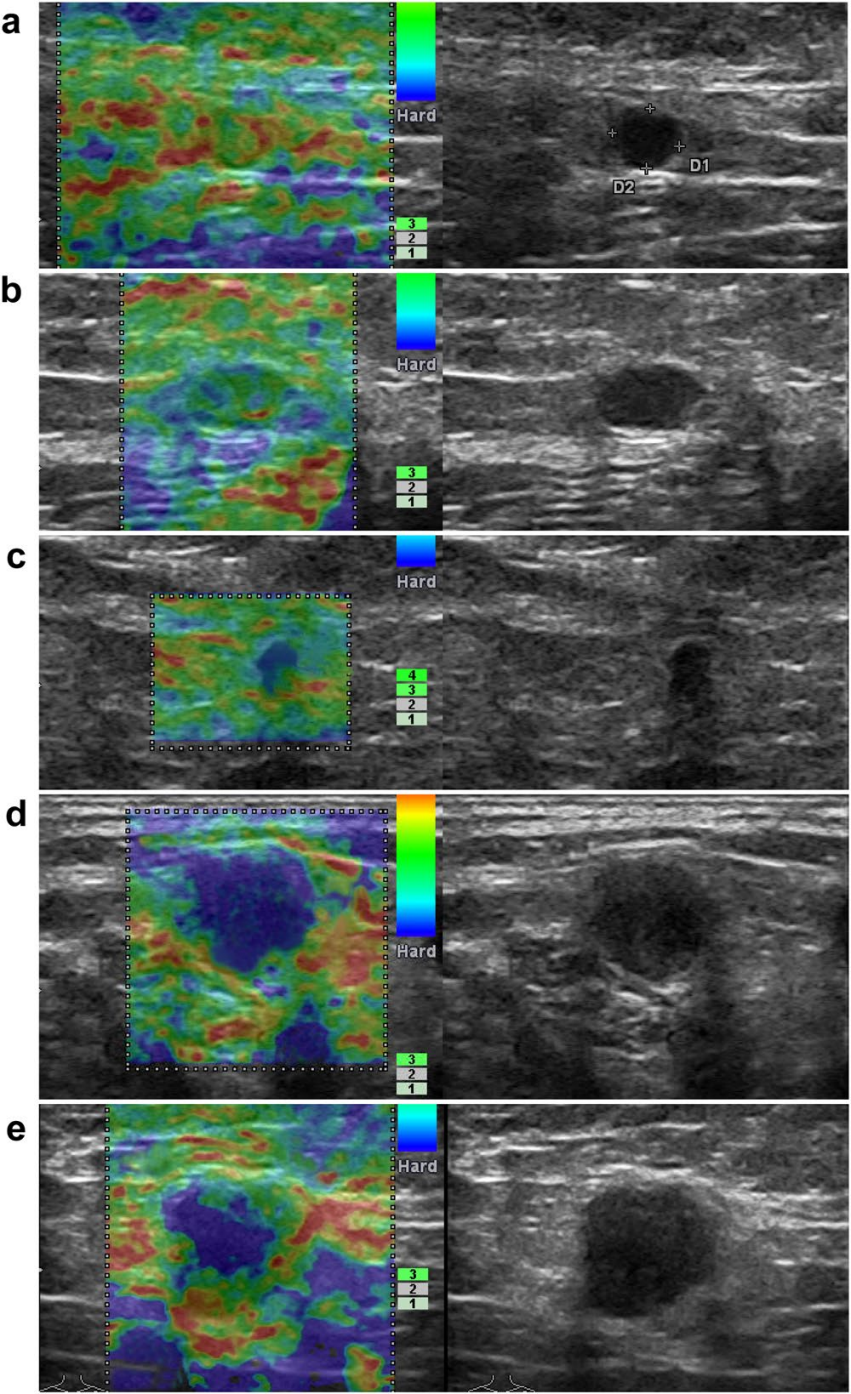
Grey scale-RTE dual-mode ultrasonogram of Pattern II ALNs. Images present the general appearance of lesions for RTE scores of (**a**) 1, (**b**) 2, (**C**) 3, (**d**) 4, and (**e**) 5 of the lymph nodes without hila: (**a**) Green portion occupying almost all of the cortex; (**b**) Blue portion occupying less than 50% of the cortex; (**c**) Blue portion occupying more than 50% of the cortex, with a scattered green/red portion; (**d**) Blue portion occupying almost all of the cortex; (**e**) Blue portion occupying almost all of the cortex, with a green/red ring on the edge of the node.

In our study, RTE analysis of the percentage and distribution of the relatively stiffer tissue focused on the hypoechoic regions in the lymph nodes. As is shown in Table S1, RTE scores were reported on a scale of 1 to 5. The pattern of the elastograms of the lymph nodes were divided into two groups: those with hila (Pattern I) (Figs 1 and 2) or those without hila (Pattern II) (Figs 3 and 4). According to the ROC curve analysis, the cutoff value was between 2 and 3 for elastography ultrasound. The nodes that were scored 1 or 2 were considered benign, and those that were scored 3, 4 or 5 were considered metastatic based on elastography. The sensitivity, specificity, PPV, NPV and accuracy were 78%, 93%, 93%, 79% and 86% respectively. RTE had a higher specificity (93% vs 73%, *P* = 0.012) and a lower sensitivity (78% vs 92%, *P* = 0.039) than gray-scale ultrasonography. Of all the 45 benign axillary lymph nodes analysed in the present study, 11 were determined to be metastatic by gray-scale ultrasonography, while these diagnoses were corrected by RTE. Seven of these 11 nodes (63.6%) were diagnosed as highly hyperplastic based on pathological analysis, and the 4 (36.7%) nodes were found to be normal.

### Combined Diagnostic Characteristics

The cutoff value was set between 5 and 6 for the combined diagnosis according to the ROC curve analysis. In the combined diagnostic group, scores from 3 to 5 were considered benign while scores from 6 to 10 were considered metastatic. The sensitivity, specificity, PPV, NPV and accuracy for combined diagnosis were 88%, 96%, 96%, 88% and 92% respectively (Table 2).

### Strain Ratio Characteristics

SR was calculated from the lesion and comparable lateral fatty tissue (Fig. S2). The mean value of SRs for benign nodes and malignant nodes were 2.35 ± 1.80 (range, 0.51 to 6.31) and 12.64 ± 5.30 (range, 7.6 to 39.9), respectively. A significant difference in strain ratio between the metastatic and the benign nodes was found according to the result of *t*-test (*t* = 8.412, *P* < 0.01). The sensitivity, specificity, PPV, NPV and accuracy for strain ratio were 87%, 76%, 80%, 83% and 81%, respectively, with the best cutoff value of 2.62 according to the ROC curve analysis (Table 2).

### Diagnostic Performance

The diagnostic performance of each parameter of gray-scale ultrasound, real-time elastography, combined evaluation and strain ratio are summarized in Table 2. Gray-scale ultrasound had a better sensitivity than RTE (92% vs 78%, *P* = 0.039), while the specificity of RTE was superior to that of gray-scale ultrasonography (93% vs 73%, *P* = 0.012).

**Figure 5:**
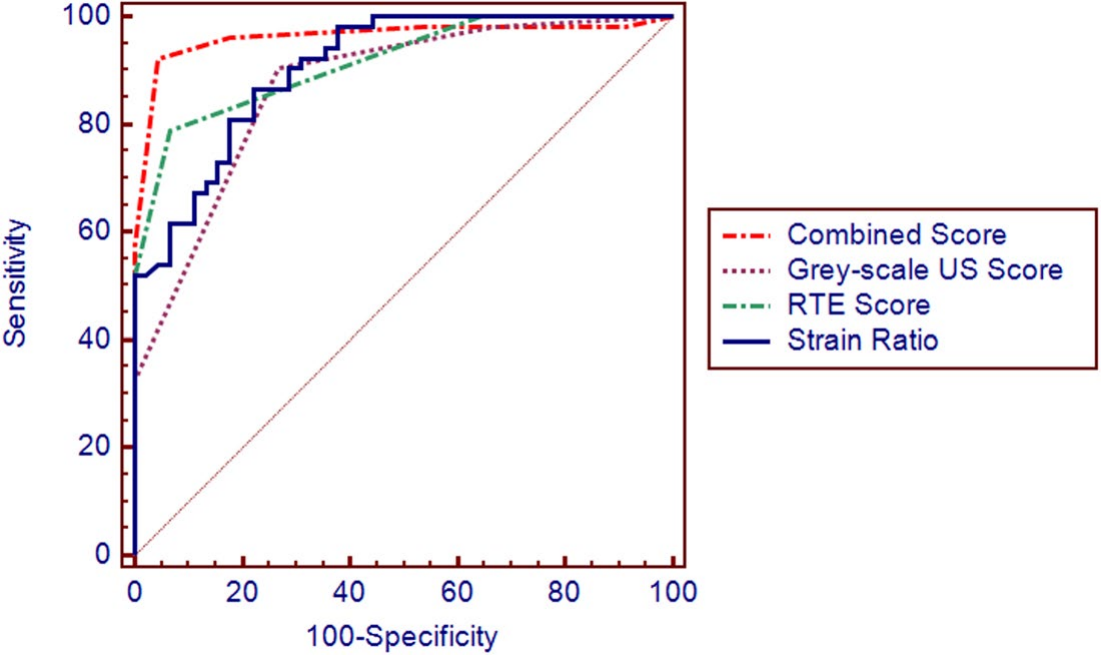
ROC curves of gray scale ultrasound, RTE, combined evaluation, and SR. Areas under the ROC curve for gray-scale ultrasound, RTE, combined evaluation and SR were 0.871, 0.916, 0.963 and 0.906 respectively. The diagnostic power of the combined evaluation was better than that of gray-scale ultrasonography (*Z* = 3.604, *P* < 0.01) and RTE (*Z* = 2.008, *P* = 0.045). The diagnostic power of gray-scale ultrasound was not significantly different compared with that of RTE (*Z* = 1.162, *P* = 0.245). The combined evaluation had better diagnostic power than that of strain ratio, but there was no significant difference (*Z* = 1.642, *P* = 0.1006).

Figure 5 shows the receiver operating characteristic (ROC) curves for gray-scale ultrasonography, real-time RTE, combined evaluation, and SR. The areas under the ROC curve for each of these techniques were 0.871, 0.916, 0.963 and 0.906, respectively (Table 3). The diagnostic power of combined evaluation was better than that of gray-scale ultrasonography (*Z* = 3.604, *P* < 0.01) or RTE (*Z* = 2.008, *P* = 0.045). The diagnostic power of gray-scale ultrasound was not significantly different compared with that of RTE (*Z* = 1.162, *P* = 0.245). There was no statistically significant difference between the AUC of strain ratio and the AUC of the combined evaluation (*Z* = 1.642, *P* = 0.1006).

**Table 3:** Analysis of the areas under the ROC curves for each diagnostic method.

Diagnostic Method	AUC	Standard Error	*P* value	CI (95%)	
Gray-scale ultrasound	0.871	0.036	<0.001	0.801	0.941
Real-time elastography	0.916	0.027	<0.001	0.863	0.969
Combined evaluation	0.963	0.021	<0.001	0.922	1.000
Strain ratio	0.906	0.028	<0.001	0.850	0.961

## Discussion

A considerable number of studies have been documented the preoperative evaluation of axillary lymph nodes status in breast cancer using gray-scale ultrasonography. However, the accuracy of conventional gray-scale ultrasonography is moderate.

Numerous published studies have analyzed the diagnostic performances of the size, long to short axis (L/S) ratio, cortex thickness, border and hilum features of ALNs as part of lymph node status evaluation. These five criteria were also adopted in the present study to evaluate the lymph node status. Our results also verified their value in the assessment of the lymph nodes, but there were still some discrepancies. Cortical thickness possessed a relatively high specificity, and the border and H/L possessed a relatively low sensitivity, which was not satisfactory. However, compared to previous reported results^8,11,14^, when using 2 as the threshold value, the long-to-short axis (L/S) ratio was not significantly different between the benign and metastatic lymph nodes (*P* = 0.066), which was consistent with the results of some studies that indicated that the L/S ratio was one of the less valuable parameters for lymph node evaluation^12,13,15,16^. The strength of the ultrasound assessment of the L/S ratio tended to be related to the small size of the nodes. H/L is a relatively new parameter proposed by Nori^27^ that has rarely been studied before. The accuracy of this parameter was quite encouraging in Nori’s study, but it was not so convincing in our study. The sensitivity of H/L was 69%, the specificity was 78%, and the accuracy was 70%. We suggest that more studies should be performed to research cutoff values for H/L for reference. We came to the consensus that no single parameter could achieve an encouraging diagnostic performance. With a combination of these parameters, a scoring system for the gray-scale ultrasonography showed an obviously high diagnostic accuracy of 83%.

Previous studies of the lymph nodes have applied an elastography pattern scoring system that is based upon the percentage and distribution of the harder tissue as a semi-quantitative method^19–26^. This method is widely used in the real-time elastography evaluations of superficial glandular organ neoplasms especially in the breast and thyroid, which have relatively homogenous tissue composition. However, this kind of scoring system assumes that the tissue constitution of lymph nodes is also homogenous. The proportion and distribution of the stiffer parts of the whole lymph nodes were observed or calculated, but the fundamental histological difference between the hilum and the cortical region was not taken into account.

The elastography scoring system used in this study is also based on the elastogram patterns proposed by Alam *et al*.^21^. However, we improved the previous system by considering the differences in the histological constitution between the hilum and cortex, as well as the pathological process of tumour lymphatic metastasis. Tumour metastasis in the lymph nodes eventually progresses in a centripetal fashion. The tumour cells drain into the lymph node through the afferent lymphatic vessel, infiltrate into the marginal sinuses, accumulate in the cortical region, pass through the lymphoid follicles up to the capsule and continue to infiltrate into the medulla until the whole lymph node is occupied by the tumour cells. This series of pathological changes that can be observed by the gray-scale ultrasonography. The cortex of the metastatic lymph nodes is thicker than that of benign nodes, and the borders of these nodes are deformed. The hyperechoic hilum region shrinks or totally disappears when the medullary sinuses are packed with tumour cells. On the other hand, the inflammation-induced immunological reaction in the lymph nodes also leads to the similar echoic changes on the gray-scale ultrasonography images, which may confuse radiologists^28,29^. Therefore, our study focused on the hypoechoic regions and the percentage and distribution of the relatively stiffer tissue in the lymph nodes during the RTE analysis.

Similar to the published studies, false negative results and false positive results could not be avoided. The false negative rates were 8.3% (4/52) and 13.5% (7/52) respectively for gray-scale ultrasound and elastography, respectively, in this study. The four malignant nodes that were misjudged by the gray-scale ultrasound all had medullary hilum structures. Four of the seven malignant nodes misjudged by the elastography had medullary hilum structures, and another 3 nodes did not. In the combined analysis, 5 nodes were still misjudged because they only had evidence of micrometastasis. These 5 lymph nodes showed some similar characteristics, including a small diameter, a regular border, a visible medullary hilum structure and a score of 2 based on elastography. We considered that these malignant nodes possessed a relatively normal appearance mainly because of the micrometastasis in the nodes (metastatic deposit >0.2 mm but <2.0 mm), which could not be detected either by gray-scale ultrasound nor elastography^28,29^. In terms of the three false positive results based on elastography, we found that two nodes were at a deep position in the axillae, and their perpendicular distances from the skin were 28 mm, and 33 mm, respectively, and they were also small nodes, measuring 5.3 mm × 2.8 mm and 6.2 mm × 4.3 mm, respectively. Another lymph node measured 33.4 mm × 9.5 mm and was approximately to the length of the long axis of the probe (45 mm).

Furthermore, only 2 nodes were larger than 2.5 cm along the long axis in this study (another one was 28 mm, with a depth of 17 mm). We considered that either the volume or the perpendicular depth of location of the lymph node may influence on the signal stability of the elastography, which was also suggested by Tourasse C *et al*.^30^. The effect of these two factors together maybe more complex. However, in our study, it was not possible to perform further analysis regrading this issue because there were only 4 nodes at a depth over 2.5 cm in our study, probably because Asian women usually have a lower body weight and possess less fatty tissue in the axillary region. We have already prepared to commence a series of studies.

Strain ratio is a semi-quantitative parameter that can be obtained using the RTE technique^18,21,23,25,26^. We used the surrounding soft tissues and adipose tissue at a similar depth as a reference to obtain the strain ratio indices considering that these tissues have relatively homogenous tissue constitution and low variation among the different patients. The cutoff value for stain ratio found in our study was similar to that reported by Choi’s^26^. However, the region of interest (ROI) we chose was different from that in the study by Choi’s. In that study, the thickest portion of the node on the gray-scale sonograms was used, while we evaluated the strain ratio indices upon the deepest blue portion of the nodes, which theoretically indicated the hardest part of the nodes.

Taylor *et al*.^25^ evaluated 50 axillary lymph nodes in patients who had suspicion of breast cancer. The sensitivity and specificity for the sono-elastography were 90% and 86%, respectively, which were superior to those of gray-scale ultrasonography. The results were quite encouraging. The cutoff point of strain value was 0.65, which had a sensitivity of 100% and a specificity of 48%. Choi *et al*.^26^ utilized a similar real-time elastography equipment to perform a study on 64 axillary lymph nodes in breast cancer patients. The sensitivity, specificity and accuracy were 81%, 67% and 73%, respectively. They also used the adipose tissue as the reference to evaluate the strain value. The optimal results were obtained at the cutoff value of 2.3. In terms of the results of our study, RTE had a higher specificity than gray-scale ultrasound. On the other hand, gray-scale ultrasound had a superior sensitivity compared with RTE. The accuracy of the combined evaluation was higher than that of both gray-scale ultrasound and RTE. Gray-scale ultrasound and RTE were complementary to each other in the evaluation of axillary lymph nodes. Elastography had a lower sensitivity and higher specificity than B-mode US. Moreover, the specificity and accuracy were higher for the combination of these techniques than for both B-mode US and elastography alone.

In terms of the discrepancy in the results between our study and previously published studies, we hypothesized that in addition to the different scoring systems for the sonograms, the differences in the software used, the practical experience of the operator should be taken into account. Standard guidelines for both the ultrasonographic scanning procedure and evaluation criteria are worth of developing. The demographic and epidemiologic characteristics of the patients as well as the characteristics of the primary tumours, such as the tumour size, location, histological type, grade, presence of peripheral vascular invasion and the immunohistochemical indicators, may also affect the assessment of ALN status in breast cancer^31,32^. On the other hand, the precision of ultrasound-guided biopsy may differ in different facilities. Though the scoring systems that are based on the extent of the metastatic foci in the lymph nodes for both of the gray-scale ultrasound and RTE are relatively precise, micrometastatic foci are still unable to be diagnosed with neither the gray-scale ultrasound nor RTE. The consistency between the elastography evaluations and the histological results of axillary lymph nodes in breast cancer patients need to be further investigated.

A limitation of our study is that the comparison between the elastograms with pathological slices was not performed, which may have allowed direct observations of the distribution of cancer cell colonies in the nodes since all the pathological results were obtained from the core biopsies. However, with ultrasound-guided core biopsy, the node-to-node targeting was ensured. Another limitation was that the two radiologists read the B-mode ultrasound and elastography images together and may have introduced a bias.

In summary, real-time elastography is a feasible technique for evaluating the axillary lymph nodes status in breast cancer patients. Our present study results showed that real-time elastography and gray-scale ultrasound may play complementary roles in the preoperative evaluation of axillary lymph node status for breast cancer patients. The combination of gray-scale ultrasound and elastography may improve the diagnostic accuracy for assessing ALN status, especially for the lymph nodes with visible hila.

## Methods

### Patients

The study was a prospective study approved by the Ethics Committee of Shanghai Jiao Tong University Affiliated Sixth People’s Hospital. From July 1^st^, 2014, to May 30^th^, 2015, 104 consecutive patients who were enrolled in the study. All the included patients were women. Written informed consent was obtained from all the enrolled patients. All the examinations were carried out in accordance with the standard protocol approved by Shanghai Jiao Tong University. All patients were treated according to the clinical guidelines of Shanghai Jiao Tong University. The inclusion criteria were as follows: This was a prospective study and consecutive patients who were referred for sonography of breast masses were enrolled. Patients with suspected axillary lymph node involvement and breast cancer were enrolled in this study. The exclusion criteria were as follows: a) history of neoadjuvant therapy; b) ductal carcinoma *in situ;* c) benign breast lesion; or d) the suspected axillary lymph node located adjacent to the main axillary vessels so that it would be risky to perform a biopsy (Fig. S1).

All patients underwent ultrasound-guided core biopsy within 3 days after the examination of the gray-scale ultrasound and RTE examinations of the breast and axillary area. Seven patients who had the medical histories of neoadjuvant therapy, 3 patients who were diagnosed with ductal carcinoma *in situ*, and 2 patients who were diagnosed with benign masses were excluded from the final analysis. Primary tumour removal and axillary lymph node dissection were performed in the other 92 patients who were confirmed to have malignant breast cancer based on tumour biopsy histological findings.

The histological types were identified as invasive ductal carcinoma in 81 patients (88.0%), invasive lobular carcinoma in 8 patients (8.7%), medullary carcinoma in 2 patients (2.2%), and mucinous carcinoma in 1 patient (1.1%).

Among the 108 axillary lymph nodes, 11 lymph nodes were anatomically adjacent to the main axillary vessels. Considering the potential risk of core biopsy for these lymph nodes, these 11 nodes were ultimately excluded from the study. Finally, 97 lymph nodes (metastatic, n = 52; benign, n = 45) from 92 patients whose mean age was 51 y (range, 25 to 79 y) were enrolled in the final analysis. The reference standard for the final diagnosis was core biopsy pathological findings.

### Image acquisition and analysis

One radiologist who had ten years’ experience in conventional ultrasonography and five years’ experience in the real-time elastography of superficial tissues performed the examinations. Gray-scale ultrasound and real-time elastography were performed using a digital ultrasound scanner (Hivision 900, Hitachi Medical Cooperation, Tokyo, Japan), equipped with a 7.5–13 MHz liner transducer.

Only the ipsilateral axillary region was evaluated with ultrasound scan. Gray-scale ultrasonography was performed first. The optimum frame for visualization of the suspicious lesion was captured on the monitor, and then the scanner was switched to the elastography mode.

Rhythmic compressions were produced in the ipsilateral axillary region with the same free-hand probe along the axis. An elastogram that was considered to be stable and reliable was stored only when the following conditions were met. First, the region of interest (ROI) included the entire node and the surrounding tissues with the same or a greater proportion of surrounding tissues to the node, so that the ROI was greater than or equal to 2 times larger than the node. Second, the compression was not considered reliable until a value of 3 or 4 on a scale of 1–5 was registered on the scanner panel, which is a comprehensive indicator of the compression pressure and frequency. Third, the colour distributed in the ROI had to remain constant for at least 5 seconds while the colour was maintained nearly at the same size and location. For each lymph node, the 3 most reliable elastograms were stored for the final analysis.

Another two radiologists who were both blinded to the medical information of all these enrolled patients evaluated the gray-scale ultrasound and elastography images. When a disagreement occurred during the evaluation, the final decision was agreed upon after discussion.

### Gray-scale ultrasound evaluation

We used a gray-scale ultrasound scoring system based on five criteria including the short-axis diameter (S >7 mm, score of 1; S ≤7 mm, score of 0), the long-to-short axis diameter ratio (L/S <2 score of 1; ≥ 2, score of 0), the hilum long axis-to-node long axis diameter ratio (H/L <0.5, score of 1; H/L ≥ 0.5, score of 0), the border (irregular, score of 1; regular, score of 0) and the cortical thickness (T >3 mm score of 1; T ≤ 3 mm, score of 0) (Table 1). When the node did not have a hilar region, the cortical thickness was regarded as ≥ 3 mm when the short-axis diameter was more than 3 mm. The total score of each lymph node was determined by the sum of all scores from the five criteria.

### Real-time elastography evaluation

After performing gray-scale ultrasound, RTE was performed. The pattern of the elastograms of the lymph nodes were divided into two groups: those with hila (Pattern I) or those without hila (Pattern II). According to the distribution and percentage of blue in the hypoechoic cortical region in the lymph nodes, RTE scores were reported on a scale of 1 to 5 as follows: 1, the green portion occupied almost all of the cortex; 2, the blue portion occupied less than 50% of the cortex; 3, the blue portion occupied more than 50% of the cortex, with scattered the green portions; 4, the blue portion occupied almost all of the cortex; 5, the blue portion occupied almost all of the cortex, with a green ring on the edge of the node. (Figs 1–4 and Supplementary Table S1).

### Combined evaluation of gray-scale ultrasound and elastography

The score of combined evaluation for each lymph node was the sum of the gray-scale ultrasound and RTE scores.

### Strain ratio

Stain ratio (SR) was measured on a static image including B-mode ultrasound and elastographic images (Fig. S2). Then, we calculated upon the deepest blue portions of the hypoechoic regions within the nodes (ROI A), which indicated the hardest parts of the nodes. We used the surrounding soft and adipose tissue at the same depth as a reference (ROI B) to obtain the strain ratio. Strain ratio is a unit-less semi-quantitative value representing the ratio between two different strain values (strain ratio = strain value of ROI B/ strain value of ROI A). The SR was automatically calculated and displayed on the monitor by the software built into the equipment.

### Pathological analysis

Ultrasound-guided core biopsy was performed by the same experienced radiologist for all lymph nodes with a semi-automated biopsy gun and the 14-G Tru-cut needles (Precisa, Hospital Service, Rome, Italy) under local anaesthesia using 5 mL of subcutaneous lidocaine. Three samples were obtained from each node at a length of 23 mm. Then, the samples were fixed in 10% formalin and sent to the pathology department immediately. The final pathological diagnosis for the lymph nodes was made by a pathologist with 15 years’ experience with the diagnosis of axillary lymph nodes who was blinded to the ultrasound findings.

### Statistical analysis

SPSS 17.0 software package (SPSS, Inc., Chicago, IL, USA) and MedCalc for Windows, version 12.1.3.0 (MedCalc Software, Mariakerke, Belgium) were used for the statistical analysis. Wilcoxon’s rank sum test or Student’s t-test was used for identifying statistically significant differences. The precision of the diagnostic parameters including the sensitivity, specificity, positive predictive value (PPV), negative predictive value (NPV) and accuracy was expressed using 95% CIs. The areas under the receiver operating characteristic (ROC) curves (AUC) were calculated and compared using the *U* test. All cutoff values were determined depending on the best accuracy identified by ROC curves. Statistical significance was assumed at *P* < 0.05 for all tests.

### Data availability

All data generated or analysed during this study are included in this published article (and its Supplementary Information files).

## Electronic supplementary material

Supplementary Information
