# Supplementary material for: Application of Real-time Elastography Ultrasound in the Diagnosis of Axillary Lymph Node Metastasis in Breast Cancer Patients
Source: Sci Rep. 2018 Jul 6;8:10234. doi: 10.1038/s41598-018-28474-y (PMC6035225; doi:10.1038/s41598-018-28474-y)
Supplement: Supplementary file 1 — Supplementary Information [file 41598_2018_28474_MOESM1_ESM.doc]

**Application of Real-time Elastography Ultrasound in the Diagnosis of Axillary Lymph Node Metastasis in Breast Cancer Patients**

Yanjun Xu^1^, Xiaojun Bai^1^, Yini Chen, Lixin Jiang, Bing Hu, Bin Hu*, & Li Yu*

Department of Ultrasound in Medicine, Shanghai Jiao Tong University Affiliated Sixth People's Hospital, Shanghai Institute of Ultrasound in Medicine, Shanghai 200233, China.

*Corresponding authors:

Department of Ultrasound in Medicine, Shanghai Jiao Tong University Affiliated Sixth People's Hospital, 600th Yishan Road, Shanghai 200233, China. Email: yuli0122@126.com (L. Yu), [niuniu1213@qq.com](mailto:niuniu1213@qq.com) (B. Hu). Tel: +86 02164369181; Fax: +86 021-64701361.

^1^These authors contributed equally to this work.

**Supplementary Table S1. Elastogram schematic presentations and scoring system of axillary lymph nodes appearance on real-time elastography**

| Score | Description | Elastogram schematic presentation | |
| --- | --- | --- | --- |
|  |  | Pattern Ⅰ^a^ | Pattern Ⅱ |
| 1 | Green portion occupied almost all of the cortex in pattern Ⅰ or the whole lymph node in pattern Ⅱ | Fig. 1a, 2a | Fig. 3a, 4a |
| 2 | Blue portion occupied less than 50% of the cortex in pattern Ⅰ or the lymph node in pattern Ⅱ | Fig. 1b, 2b | Fig. 3b, 4b |
| 3 | Blue portion occupied more than 50% of the cortex in pattern Ⅰ or the lymph node in pattern Ⅱ, the green/red portion localizes scattered | Fig. 1c, 2c | Fig. 3c, 4c |
| 4 | Blue portion occupied almost all of the cortex in pattern Ⅰ or the lymph node in pattern Ⅱ | Fig. 1d, 2d | Fig. 3d, 4d |
| 5 | Blue portion occupied almost all of the cortex in pattern Ⅰ or the lymph node in pattern Ⅱ, with a green/red ring on the edge of the node | Fig 1e, 2e | Fig. 3e, 4e |

Note－Pattern Ⅰ including the lymph nodes with visible hila, Pattern Ⅱ including the lymph nodes with absent hila.


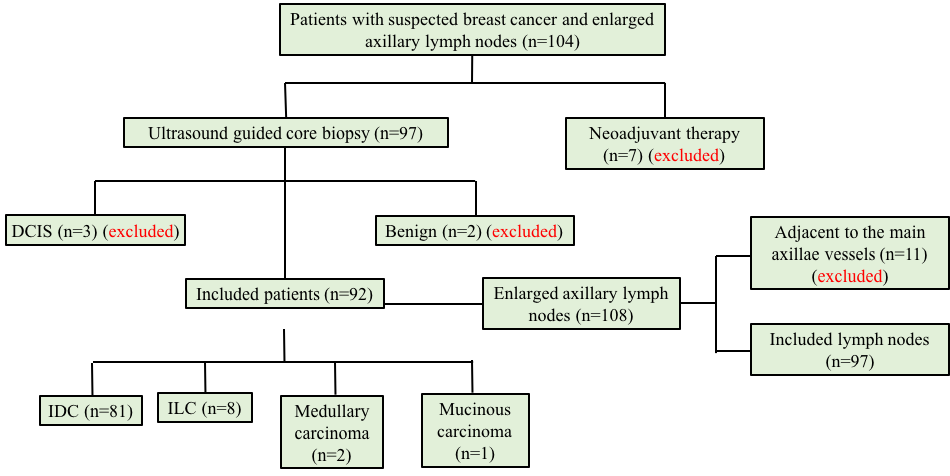


Figure S1. Patients enrolled according to inclusion and exclusion criteria.


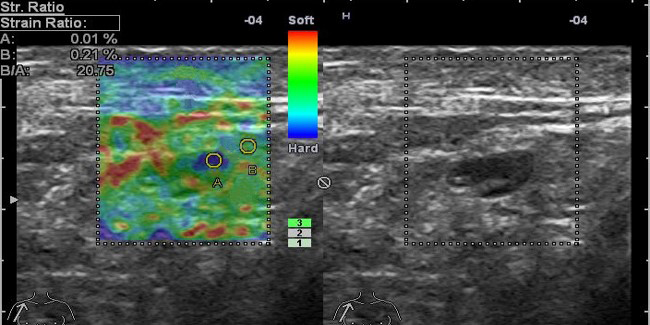


Figure S2. Strain ratio calculation illustration.
